# Olfactory phenotypic expression unveils human aging

**DOI:** 10.18632/oncotarget.8393

**Published:** 2016-03-26

**Authors:** Andrea Mazzatenta, Alessandro Cellerino, Nicola Origlia, Davide Barloscio, Ferdinando Sartucci, Camillo Di Giulio, Luciano Domenici

**Affiliations:** ^1^ Department of Neuroscience, Imaging and Clinical Science, ‘G. d'AnnunziO' University of Chieti-Pescara, Chieti, Italy; ^2^ Scuola Normale Superiore, Pisa, Italy; ^3^ Neuroscience Institute, CNR-Pisa, Pisa, Italy; ^4^ Department di Medicina Clinica e Sperimentale, Sezione di Neurologia, e Dai di Neuroscienze, Pisa, Italy; ^5^ Department of Applied Clinical Science and Biotechnology (DISCAB), School of Medicine, l'Aquila University, L'Aquila, Italy

**Keywords:** aging, olfaction, olfactometry, absolute threshold, olfactory phenotype, Gerotarget

## Abstract

The mechanism of the natural aging of olfaction and its declinein the absence of any overt disease conditions remains unclear. Here, we investigated this mechanism through measurement of one of the parameters of olfactory function, the absolute threshold, in a healthy population from childhood to old age. The absolute olfactory threshold data were collected from an Italian observational study with 622 participants aged 5-105 years. A subjective testing procedure of constant stimuli was used, which was also compared to the ‘staircase’ method, with the calculation of the reliability. The n-butanol stimulus was used as an ascending series of nine molar concentrations that were monitored using an electronic nose. The data were analyzed using nonparametric statistics because of the multimodal distribution. We show that the age-related variations in the absolute olfactory threshold are not continuous; instead, there are multiple olfactory phenotypes. Three distinct age-related phenotypes were defined, termed as ‘juvenile’, ‘mature’ and ‘elder’. The frequency of these three phenotypes depends on age. Our data suggest that the sense of smell does not decrease linearly with aging. Our findings provide the basis for further understanding of olfactory loss as an anticipatory sign of aging and neurodegenerative processes.

## INTRODUCTION

Olfaction, or the sense of smell, is devoted to the capture of the infinite molecular diversity of the environment, to extract vital information through the generation of individual perceptions [1] that relate to food, in particular, and to our surroundings and relationships. Functional impairment of olfaction has a negative impact on quality of life, and on health and socioeconomic consequences. Olfaction is a chemosensory processing system that can detect potentially infinite numbers of low-molecular-mass compounds, known as odorants, which combine at different concentrations to elicit this complex perception.

Recently, there has been discussion around the magnitude of olfaction and its subjective capabilities. In addressing these questions, the lower limit of human olfactory discrimination has been increased to more than one ‘trillion’ (10^12^) odors [1]. Furthermore, the existence of an individual olfactory fingerprint has been clarified, and this relates to the expression of a unique subset of the repertoire of olfactory receptors that appears to be genetically related to human leukocyte antigen [2]. Despite these breakthroughs, the question of how olfaction fluctuates over time remains largely unanswered, and in particular, the process of natural aging of olfaction and its decline in the absence of any overt disease conditions [3-5].

To define this process of natural aging and the decline of olfaction, we measured one of the olfactory functional parameters across the full spectrum from children to the elderly. As the olfactory function is structured in a multidimensional stimulating/ perceptual space, its evaluation is feasible through its disassembly into its principal physiological components, in terms of its sensory threshold, discrimination, and identification [6, 7]. Here we measured the absolute olfactory threshold as defined by Doty and Laing [8], as “the lowest odorant concentration where the faint presence of an odor is distinguished”.

We show here that the age-related variations in the absolute olfactory threshold are not continuous; instead, there are multiple olfactory phenotypes. Three distinct age-related phenotypes are defined, which we have termed the ‘juvenile’, ‘mature’ and ‘elder’ olfactory phenotypes.

## RESULTS

### Absolute olfactory threshold evaluation

**Figure 1:**
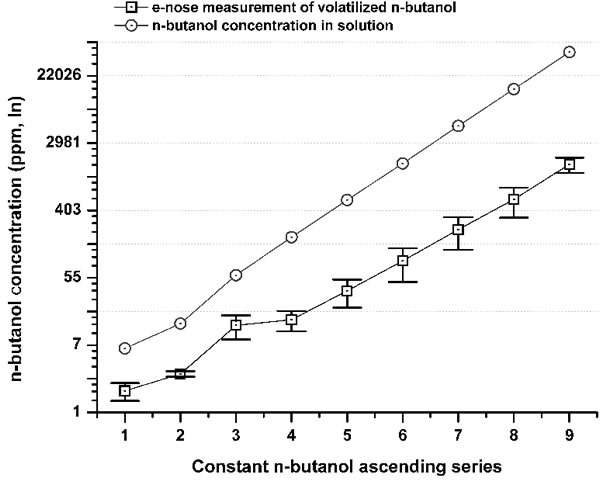
Comparison between n-butanol concentrations (ppm) in a solution series (gray circle) and its volatilized concentration measured by the e-nose (iAQ-2000; ppm) (black squares) The differences (Δ) between the concentrations (ppm) in the solution series and their volatilized fraction from #1 to #9 were: 71.7%; 77.6%; 77.5%; 91.4%; 93.3%; 94.5%; 95.4%; 96.2%; 96.4%. The overall mean Δ was 88.2% ±9.7 SD. The Δ highlights a tremendous bias, which is independent of the method or the reference material. By measuring the exact amount of the volatilized n-butanol from the stimulating solution, the absolute olfactory threshold was referred to the real stimulus concentration that reached the nose. The experiments, e-nose recordings, and testing were performed under controlled conditions, with the temperature kept at 23°C.

The absolute olfactory threshold is the lowest odorant concentration at which the presence of an odor can be distinguished [8]. This threshold was measured here by olfactory testing on a healthy population (*n* = 622; age range, 5-105 years). We also reduced the chemo-physical variability by using an electronic nose device (e-nose) to measure the differences (Δ) between the measured quantitative curves of the volatilized reference material and the respective stimulation solutions in real time (Figure 1). This represents the most important bias that is often ignored in olfactory psychophysiological testing. The reference material applied was that which is in widest use, n-butanol [8, 9, 14], and its dilutions (see Methods).

To provide an experimental basis for the method of olfactory perception used here, we initially compared the two most used methods, as those of constant and ‘staircase’ stimuli [8]. When these were compared across a randomly selected subgroup (*n* = 20; equally distributed according to gender), no significant differences emerged (F_(1,18)_ = 0.43; *p* = 0.51). Thus, the choice here was to use the standard method of constant stimulus, to avoid the other potential bias that can arise from the physiological mechanism of adaptation.

In addition, we performed another control, as indicated by Doty et al. [13]: the test was repeated with a further random subgroup (*n* = 20), to calculate the reliability coefficient (0.82). Here, repeated measure ANOVA revealed no significant main effects for the factors of ‘test/ re-test’ (F_(1,18)_ = 0.39, *p* = 0.54) and ‘gender’ (F_(1,18)_ = 0.64, *p* = 0.44), nor did these show any significant interactions (F_(2,38)_ = 0.005, *p* = 0.94).

### Absolute olfactory threshold phenotyping

**Figure 2:**
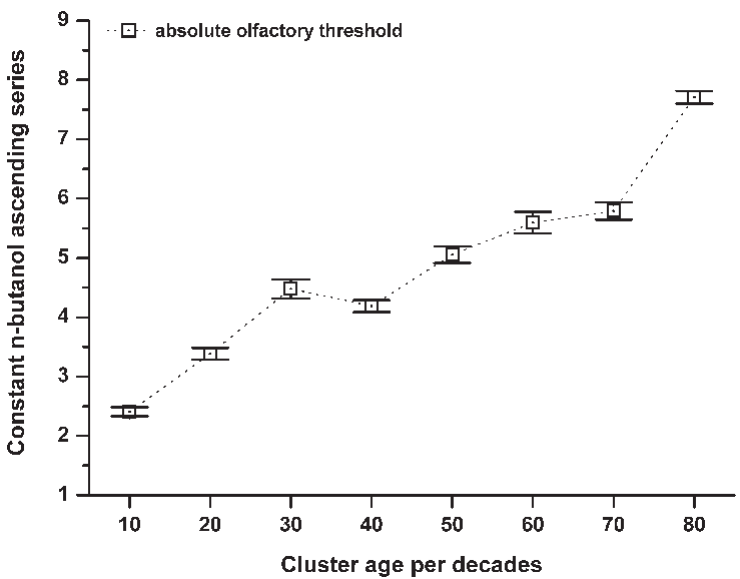
Variation in the absolute olfactory threshold aging Relationship between the constant n-butanol ascending series (see Figure 1) and the cluster ages per decade: 10 (*n* = 18); 20 (*n* = 291); 30 (*n* = 127); 40 (*n* = 39); 50 (*n* = 55); 60 (*n* = 47); 70 (*n* = 29); > 80 (*n* = 16). Data are means ±standard deviation. The mean score shift increased within aging, which corresponds to a decrease in the absolute olfactory threshold.

**Figure 3:**
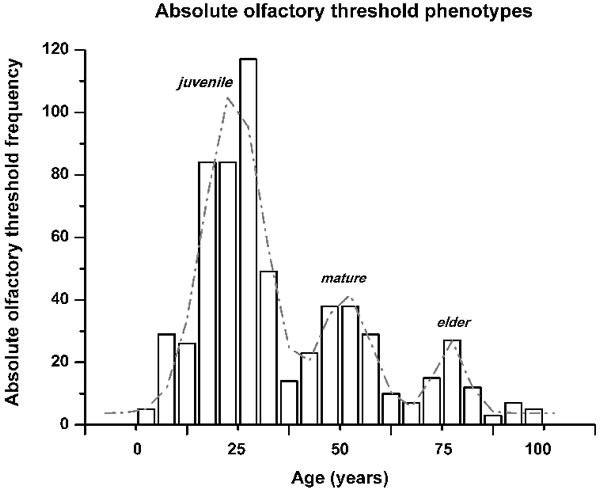
Olfactory phenotype identification by the absolute olfactory threshold frequency distribution across the ages of the subjects The distribution obtained was not continuous. The fit of the distribution indicates three peaks, with R^2^ = 0.92, and a reduced Chi^2^ of 124.7, which are termed here as: ‘juvenile’, from frequencies range from n-butanol concentration #1 up to #3.4; ‘mature’ from #3.5 up to #5.9; and ‘elder’ as greater than #6.

To define the progression of the absolute olfactory threshold through the human life span, the data were clustered into age decade classes (Figure 2), according to similar methods reported in the literature [8, 13]. This demonstrated that the sense of smell as assessed by this determination of the absolute olfactory threshold progressively declined with age (Figure 2). This absolute olfactory threshold did not show a unimodal distribution (Kolmogorov-Smirnov normality test, *p* = 0.17), but instead showed a multiple peak distribution, which discriminated three age-related phenotypes (non-parametric Kruscal-Wallis test, α = 0.05, Chi-square = 531.2). Consequently, these groups were termed ‘juvenile’, from the initial n-butanol dilution #1 up to #3.4; ‘mature’, from #3.5 up to #5.9; and ‘elder’, as greater than #6 (Figure 3). There were no statistically significant differences between the sexes (non-parametric Kruscal-Wallis test, α = 0.05, Chi-square = 2.4), with both expressing the three phenotypes.

### Absolute olfactory threshold aging

**Figure 4:**
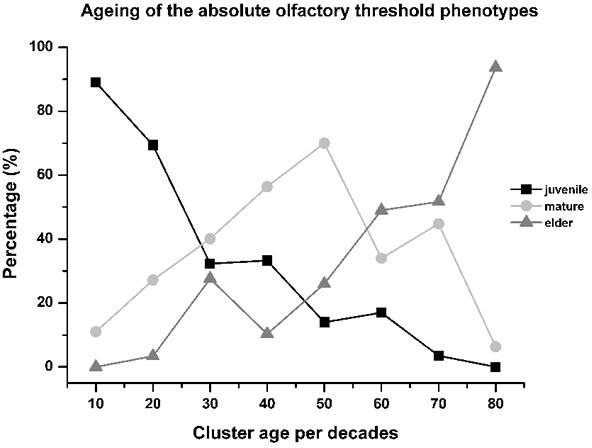
Aging of the absolute olfactory threshold phenotype The relative levels of juvenile phenotype decreased by about 60% within the third decade of life, contemporary with the increase to 25% of the subjects with the mature and elder phenotypes, which appears anticipatory of olfactory decline. From the 30 years to 60 age years clusters, the aging processes were manifest with the maximum expression of the mature phenotype (70% of subjects in the 50-years-old cluster), while the relative proportions of the juvenile phenotype decreased slowly and that of the elder phenotype started to increase, with both roughly linear during this period. For the last age clusters (i.e., up to 80 years old), the relative proportions of the juvenile and mature phenotypes were less than 10%, with the elder then the most represented phenotype (> 90%). This indicates that the olfactory phenotype of aging can start to develop in young individuals even before sexual maturity. As expected, the juvenile phenotype progressively decreases as age increases.

A further aspect defined here is that the absolute olfactory threshold declined with aging, as follows: the proportion of subjects with the juvenile phenotype decreased almost linearly through life, with a mirror increase in the elder phenotype. Instead, the proportion of subjects with the mature phenotype showed a bell-shaped curve, with the peak in the 50-year-old age cluster (Figure 4).

The frequencies of these three different olfactory phenotypes (i.e., juvenile, mature, elder) depend on age, as about 60% of the subjects lost their juvenile phenotype within the third decade of life, with its preservation to an elderly age in about 30% (Figure 4, see black line). Conversely, during this lifespan segment, about 25% of the total subjects developed the mature and elder olfactory phenotypes (Figure 4, see grey lines).

## DISCUSSION

This analysis shows that age-related variations in the absolute olfactory threshold are not continuous; instead, there are multiple olfactory phenotypes, a situation that has not been described previously. These data thus define three distinct age-related phenotypes, which we have termed the ‘juvenile’, ‘mature’ and ‘elder’ olfactory phenotypes.

We investigated here the absolute olfactory threshold for an odorant, namely n-butanol. The basis for the choice of any specific odorant, including n-butanol [15, 16], can be debated for a number of reasons, such as variability in the chemical and physical nature, concentrations used, subjective anosmia or dysosmia, and cross-modal stimulation. The e-nose measures as applied here removed the physicochemical factors that affect odorant volatility, thus reducing the experimental variability for the determination of the absolute olfactory threshold.

The first main result here is that absolute olfactory threshold decay does not show a unimodal distribution, but instead shows a multiple peak distribution, which highlighted the three age-related phenotypes, as juvenile, mature and elder. These data can thus explain the controversial reports that have previously overlooked the existence of multiple olfactory phenotypes [13, 17-20], which were based on the postulate that aging of olfactory function follows a progressive linear decline due to structural/ functional changes that occur to the aging nose and olfactory system. This also followed the common belief that aging is caused by random accumulation of molecular damage due to failure of repair [13, 20-26]. In contrast, olfactory aging is not a linear mechanism. Furthermore, this result is in line with the ‘hyper-function theory’ suggesting that cellular hyper-functions cause primary loss of homeostasis, which is the essence of aging; secondary are age-related diseases, for instance decline of functions, malfunctions, atrophy and damage, which is a macro event, is caused by aging, not the reverse [26]. The presence of these three phenotypes suggests that the decline of a given function, or its malfunction or damage (which is not molecular, but macro damage to a tissue, system or organ), are secondary effects of aging.

These data were also analyzed in terms of the frequency distributions of the three phenotypes, which indicated that some young individuals showed the elder phenotype. This would appear to be a sign of aging and to represent a risk factor for decline and damage in these subjects, who might be highly exposed, for example, to neurodegenerative diseases [26]. Consequently, the aging of the olfactory function is crucial for the loss of homeostasis, and hence its onset in younger subjects would be predictive of earlier decline and disease.

The second main result is that the frequencies of these juvenile, mature and elder olfactory phenotypes depend on age, as about 60% of the subjects lost their juvenile phenotype within the third decade of life. Interestingly, the age-dependent normal decline of the olfactory phenotype starts in young individuals at the end of adolescence, and progressively passes through the subsequent phenotypes as the age of each subject increases, possibly in correlation with genetic polymorphisms, as an individual fingerprint [2, 17, 18], and/or due to lifestyle and environmental factors. Thus the statement that olfactory function decreases with aging is a simplistic perspective [4, 20, 25-31], as we have seen here that younger subjects can already show expression of the elder-like phenotype.

Based on the early decline of olfactory function in such young subjects, we raise the hypothesis that the aging of olfactory functions is a dynamic process, with signs of elder-linked features present even at a young age. Indeed, olfactory dysfunction might be considered as an early sign of neurodegenerative disease, such as Alzheimer disease, even before the onset of cognitive decline [31-34]. Consequently, a corollary outcome is that young individuals with an increased frequency of the elder phenotype might be considered as subjects with very early evidence of forthcoming neurodegenerative processes.

## MATERIALS AND METHODS

### Sample

Six hundred and twenty two healthy individuals (mean age, 29.66 years ±17.1 SD; age range, 5-105 years) were enrolled, as representative of the Italian geographical spread: 256 males (mean age, 30.67 years ±18.3 SD; interquartile range [Q3-Q1], 25 years; age range, 5-88 years) and 366 females (mean age, 28.95 years ±16.24 SD; interquartile range [Q3-Q1], 14 years; age range, 5-105 years). The exclusion criteria were smoking, alcohol or narcotics consumption, impaired sense of smell, any overt pathology or disease, or recent clinical surgery or anesthesia, and the use of any drugs (including chlorhexidine). Moreover, the additional exclusion criteria for the women were during their estradiol, luteinizing hormone and follicle-stimulating hormone peaks, or pregnancy or contraceptive pill use, to prevent bias due to these physiological and pharmacological statuses. All experimental procedures were clearly explained, and the participants or their parents provided written informed consent prior to the testing sessions. The participants were free to interrupt the testing sessions at any time. The study was performed in agreement with the ethical standards of the Helsinki Declaration 2008, and the procedure was approved by the local Human Review Board (n. 3135).

### Study design

The experiments were performed under standardized conditions in a well-aired/ odorless room, without any bias, including laterality, and with the temperature set at 23°C. The olfactory stimulation was the widest used in similar studies, as n-butanol (C_4_H_10_O) and its dilutions, which were administered from a single sniffing point. The subjects were trained to report only a faint olfactory sensation, with no need for specific olfactory perception or identification, and with no somato-sensorial bias; e.g., pungency, itching, cooling or horripilation. A comparison between constant stimulation, as used here, and staircase stimulation was carried out initially on a random age sample (N = 20; 10 males; mean age, 37.9 years ±21.9 SD; 10 females; mean age, 35.7 years ±18.9 SD).

### Testing method

In the threshold tests used here, we followed the Cain test [9], with the n-butanol stimulus used as an ascending series of nine molar concentrations: (#1) 9.14 ×10^−5^ M; (#2) 2.74 ×10^−4^ M; (#3) 8.23 ×10^−4^ M; (#4) 2.45 ×10^−3^ M; (#5) 7.4 ×10^−3^ M; (#6) 2.20 ×10^−2^ M; (#7) 6.7 ×10^−2^ M; (#8) 2.00 ×10^−1^ M; (#9) 6.00 ×10^−1^ M. The dilution medium was a sterile odorless gel (FIAB, Italy) and disposable vials were used for each subject and test. The absolute threshold detection was obtained as the mean of three trials in which each subject was required to stop the test when they could identify a faint olfactory sensation (i.e., the absolute olfactory threshold).

### The e-nose

The volatilized n-butanol stimulus was measured in the real-time setting using an e-nose sensor [for methods, see 10-12] (iAQ-2000; AppliedSensor, Warren, NJ). Furthermore, the measurement of the ‘noise’ of the volatiles in the experimental room and of the dilution medium itself were both removed from the data.

### Testing controls

The magnitude of the differences between the nine n-butanol dilutions and the measures of the volatilized n-butanol from the e-nose determinations were expressed as percentages of the ppm natural logarithm values (i.e., the difference values; Δ).

To validate the testing processes and the absolute olfactory threshold test itself, the reliability was calculated. The test was thus used for another random age sample (N = 20; mean age, 28.6 years ±4.4 SD; balanced for sex: 10 males; mean age, 29. years 5 ±5.5 SD; 10 females; mean age, 27.5 years ±3.0 SD), whereby the same group of subjects was tested on two different days within a 30-day maximum interval. The results of these two tests were compared in terms of reliability coefficient and repeated measures analysis of variance.

### Age clustering

Following the testing process on the entire population, the data were clustered as age classes, with 10-year age clustering used (up to 10 years old, 11-20 years old, and so on, to > 80 years old), in agreement with the previous literature [8, 13].

### Statistics

The data analysis was performed using the MatLab, Origin, SPSS software, and the data analysis and plots were based on dilutions #1 to #9 of the molar (M) absolute olfactory threshold scale. To determine the significance of the main effects, repeated measure ANOVA was used. Kolmogorov-Smirnov normality tests and non-parametric Kruscal-Wallis tests were used to analyze the age distribution of the decreased absolute olfactory threshold scale.
